# Thromboembolic events in patients with advanced stage non-small cell lung cancer treated with platinum-based chemotherapy: a prospective observational study

**DOI:** 10.3332/ecancer.2018.876

**Published:** 2018-10-09

**Authors:** Amit Joshi, Shruti Kate, Vanita Noronha, Vijay Patil, Vaishakhi Trivedi, Supriya Goud, Sucheta More, Sandeep Bhairva, Kumar Prabhash

**Affiliations:** Tata Memorial Hospital, Mumbai 400012, India

**Keywords:** thromboembolic events (TEs), platinum-based chemotherapy, lung cancer

## Abstract

**Objectives:**

Cancer is frequently complicated by thromboembolic events (TEs). We aimed to determine the incidence of TEs in lung cancer patients treated with platinum-based chemotherapy and study patients’ baseline and treatment attributes correlating with its onset.

**Materials and methods:**

Advanced lung cancer patients started on platinum-based chemotherapy were evaluated at baseline and during routine visits for the development of TEs. The duration of follow-up was 4 weeks from the last chemotherapy. A TE occurring between the first dose of chemotherapy and 4 weeks after the last dose was considered to be chemotherapy associated.

**Results:**

Of the 165 patients on platinum chemotherapy who completed follow-up, TEs occurred in 4.8% (8 out of 165) patients. Among these, three patients had developed venous pulmonary thromboembolism and five patients had developed cerebral infarction, out of which four had arterial cerebral infarction and one patient had a superior sagittal sinus thrombosis. The majority of events (7 out of 8) occurred within 100 days of starting platinum chemotherapy. Overall, the median time until occurrence of TE was 48 days (range, 10–130 days). None of the presumed risk factors were found be associated with the occurrence of TEs on univariate analysis.

**Conclusions:**

Advanced lung cancer patients on platinum chemotherapy are predisposed to thromboembolism due to many factors. Despite its lower incidence in our study, exclusion of patients with prior thrombosis suggests the incidence of de novo thrombosis, and hence raises a valid question of the need of thromboprophylaxis in a selected group of patients.

## Introduction

Cancer is a pro-thrombotic condition and its treatment is frequently complicated by thromboembolism which adds to the overall morbidity, mortality and economics of healthcare systems [1]. Overall, thromboembolic events (TEs) occur in 4–20% cancer patients in various studies [2, 3]. A prospective study by Heit *et al* [4] has shown that although cancer alone leads to a 4.1-fold increase in thrombosis risk, chemotherapy increases that risk to 6.5-fold. In lung cancer patients, thromboembolic complications are common, with incidences ranging from 10–17 % [5–7]. Among all chemotherapeutic agents, platinum-based regimens are significantly associated with venous TEs [8]. Use of certain concomitant drugs such as steroids, granulocyte colony-stimulating factors has also been associated with an increased incidence of TEs in various studies [9]. Khorana *et al* [10] developed a risk scoring tool that can identify patients at high risk for thromboembolism using a combination of parameters. The Khorana score was recently found to be associated with the increased risk of thromboembolism in lung cancer patients in a retrospective analysis by Moore *et al* [11] among other risk factors such as sex, age, race, performance status, exposure to erythropoeitin, presence of central venous catheter, type of cancer, prechemotherapy haemoglobin and leukocyte count.

This raises a valid question of thromboprophylaxis in this group of patients. The established American Society of Clinical Oncology guidelines recommend prophylactic anticoagulation for all oncology patients in high-risk settings only. However, in ambulatory patients on chemotherapy, prophylactic anticoagulation is not currently recommended except in high-risk settings for highly selected outpatients with solid tumours receiving chemotherapy on a case-by-case basis [12]. In view of the paucity of data in lung cancer patients of Indian ethnicity, we thought that it was imperative to prospectively review the occurrence of TEs in this group so as to address the question of thromboprophylaxis in the future.

## Materials and methods

We conducted a prospective observational study comprising of patients of advanced stage non-small cell lung cancer (NSCLC) treated with platinum-based chemotherapy in Tata Memorial Hospital, Mumbai, from December 2014 to December 2016. An institutional review board approval was taken.

The primary objective of the study was to determine the incidence of TEs including deep venous thrombosis (DVT), pulmonary embolus (PE), cerebrovascular accident, and unstable angina/myocardial infarction (MI) in the study population patients of lung cancer treated with platinum-based chemotherapy.

All patients with advanced stage NSCLC (age >18 years) who were started on platinum-based chemotherapy were included after undertaking written informed consent. The following patients were excluded: (1) presence of any TE before the start of chemotherapy including DVT, PE, cerebrovascular accident and unstable angina/MI, (2) patients with bleeding diathesis, inherited coagulopathy and those requiring therapeutic anticoagulation, regular nonsteroidal anti-inflammatory drugs and aspirin, (3) patients receiving angiogenesis inhibitor drug bevacizumab. Patients who were excluded from the study were planned for therapy as per the standard guidelines as per the treating medical oncologist. Patients included in the study were evaluated at the baseline prior to the start of chemotherapy by taking a detailed history including history of smoking and concomitant drugs such as aspirin, statins and detailed physical examination. Baseline characteristics of the patients were noted including sex, body mass index (BMI), performance status, hemoglobin, total leucocyte count, platelet count and fasting lipid profile. Patients were then assigned to the Khorana risk group [10] (low, intermediate or high). All patients were enquired for the development of any TE at every visit with history pertaining to any TE. In the case of a new onset limb swelling, a venous Doppler was done to rule out DVT. If there was a new onset history of chest pain or shortness of breath, an electrocardiogram, cardiac biomarkers: Troponins T and I and myocardial muscle creatine kinase (CK-MB), were done to rule out acute coronary syndrome. PE was suspected in all patients with acute onset breathlessness and computerised tomography pulmonary angiography was done to confirm the diagnosis. In the presence of a new onset headache or a focal neurological deficit, magnetic resonance imaging of the brain was done to rule out metastases or progression and confirm the diagnosis of a cerebrovascular event. Subsequently, if the diagnosis of TE was confirmed, the patient was started on anticoagulation—either low molecular weight heparin (LMWH) followed by oral warfarin or LMWH alone as per the institutional policy and guidelines [13] for a duration of at least 3 months. Anticoagulation treatment beyond 3 months was decided on the basis of benefit-risk ratio, tolerability, patients’ preference and cancer activity. A TE was considered to be associated with chemotherapy if it occurred between the time of the first dose of platinum-based chemotherapy and 4 weeks after the last dose. Those patients, who were asymptomatic and were incidentally found to have a TE on reassessment imaging, were also started on anticoagulation at the earliest. The further platinum-based chemotherapy was withheld for all patients who developed a TE.

## Statistical analysis

Presuming the incidence of TEs to be between 10% and17%, with an alpha error of 5% and power of study of 80%, we calculated a sample size of 167 patients using the statistical analysis system software. The duration of follow-up was 4 weeks from the last dose of chemotherapy. The factors associated with thromboembolism were studied in univariate or multivariate analysis. Univariate analysis was done by chi-square test and multivariate analysis by Cox regression test.

## Results

The study was conducted over a period of 2 years from December 2014 to December 2016. A total of 188 patients were screened, out of which 21 patients were excluded for the following reasons: receiving antiplatelet drugs (*n* = 6), history of thromboembolism (*n* = 10), patient refusal (*n* = 1) and declared unfit for chemotherapy (*n* = 4).

Baseline patient characteristics are described in Table 1. The mean age of the patient population was 57.5 years (range 30–79), the majority of the patients were male and either current or former smokers. Most patients had stage IV disease and a good performance status (ECOG 0-1).

## Thrombotic risk factors

Table 2 lists the common risk factors for thrombosis which were seen amongst the study population. It was observed that the majority of the patients had a normal BMI. On classifying patients according to the Khorana score, it was observed that a majority of 129 out of 167 (77.2%) belonged to the intermediate group (Risk score 1–2).

## Treatment characteristics

Of the 165 patients, 67.8% (112/165) received a chemotherapy regime of carboplatin with gemcitabine, 30.3% (50/167) received carboplatin with pemetrexed, 1.2 % (2/165) received cisplatin with pemetrexed and 0.6 % (1/165) received carboplatin with paclitaxel. The median number of days on platinum was 94 (range 1–478). The median number of chemotherapy cycles administered was 4 (range 1–6) for both cisplatin and carboplatin-treated patients. The mean dose of carboplatin and cisplatin over the course of treatment was 1873 mg (313–4200 mg) and 300 mg/ m^2^ (150–450), respectively.

## Characteristics of observed thromboembolic events

**Figure 1.**
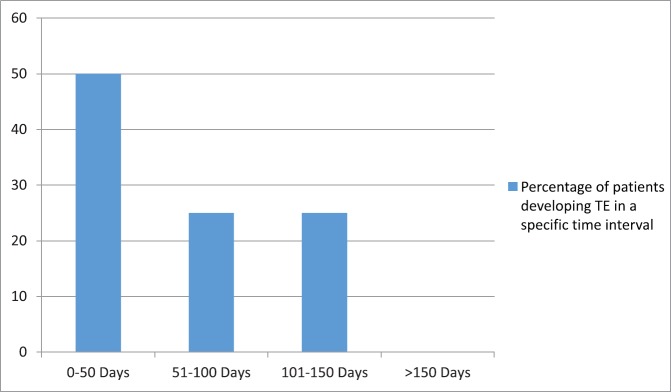
Time interval for the development of thromboembolic event (TE) from the start of platinum-based chemotherapy.

TEs related to platinum chemotherapy occurred in 4.8% of patients (8 out of 165 patients). Among these eight patients with TEs, three patients developed venous pulmonary thromboembolism and five patients developed cerebral infarction, out of which four had arterial cerebral infarction and one patient had a superior sagittal sinus thrombosis. All eight patients were symptomatic and one patient with cerebral infarction died because of the infarction. Figure 1 represents the time for the development of a TE from the start of platinum chemotherapy among patients who developed a TE. The majority of events (7 out of 8) occurred within the first 100 days of starting platinum chemotherapy. Overall, the median time until occurrence of the TE was 48 days (range, 10–130 days).

## Univariate analysis of risk factors associated with thrombosis

Table 3 shows the common risk factors for the study population, comparing patients with TEs during chemotherapy to those without thromboembolism. None of the factors amongst the following—diabetes, hypertension, smoking, BMI, hypertriglyceridemia, baseline haemoglobin, baseline leucocyte count, baseline platelet count or Khorana score—were found to have a significant association with thromboembolism in the studied population on univariate analysis. Another factor which was considered as a risk factor was the presence of a central venous catheter, but none of the patients included in the study required a central venous access.

## Discussion

In this prospective observational study, we found that in our study population of 165 patients, the incidence of TEs was 4.8%. None of the presumed risk factors associated with thrombosis were found to be related to the occurrence of TEs on univariate analysis.

In the large retrospective analysis of 932 cancer patients at the Memorial Sloan Kettering Cancer Center in 2008 by Moore *et al* [11], 24 out of 204 (11.8%) patients with lung cancer had experienced a TE during treatment or within 4 weeks of the last dose of cisplatin chemotherapy.

In another retrospective analysis in 784 patients with NSCLC treated with platinum-based chemotherapy by Mellema *et al* [14], 8% (55/665) of the patients exposed to cisplatin developed a TE versus 5% (8/171) in patients exposed to carboplatin. The difference in the incidence of TEs in patients receiving cisplatin or carboplatin was not statistically significant (*p* = 0.42).

Both the studies quoted above were retrospective analyses. Since our study was a prospective observational study, we excluded patients who had a prior history of thromboembolism including both arterial and venous TEs.

It has also been noted in the study by Mellema *et al* [14], that the only risk factor which was associated with occurrence of thromboembolism among all other factors during chemotherapy, was a prior history of thromboembolism (*p* < 0.01) suggesting that a proportion of patients analysed in this trial had a predisposition to the development of thrombosis. In comparison, in our study, the exclusion of patients with prior thromboembolism eliminates this contributory risk factor, hence establishing the incidence of de novo thrombosis in lung cancer patients who are treated with platinum-based chemotherapy.

The lower incidence of TEs in our study could be due to exclusion of patients with a prior history of thromboembolism and the use of carboplatin-based chemotherapy in the majority of patients. Although the influence of race and ethnicity on thromboembolism has been described in general, a lack of consistency in the classification of racial/ethnic groups limits the application of knowledge of ethnic variation in this particular group of patients [15].

The risk of thromboembolism in cancer patients is the highest in the first few months after diagnosis [16]. The timing of TEs (TEEs) in relation to the initiation of therapy further suggests a relation between platinum administration and TEE occurrence. In the retrospective study by Moore *et al* [11], 88% of TEEs occurred within the first 100 days of starting platinum chemotherapy. A similar finding was noted by Numico *et al* [7], in which 45% of the vascular events occurred during the first two courses of cisplatin and gemcitabine. Similarly, in a study by Weijl *et al* [17], the median time interval between initiation of platinum-based treatment and first TEE was 52 days.

In our study also, the majority of events (75%) occurred within the first 100 days of starting platinum chemotherapy. Overall, the median time until occurrence of TEs was 48 days (range, 10–130 days).

Table 4 compares the results of this study with the published data on this subject.

On univariate analysis, none of the risk factors for thrombosis were found to be statistically related to the occurrence of TEs. This could be due to the small number of events and sample size in this study.

Although the low incidence of TEs in this study does not justify the routine use of thromboprophylaxis in all lung cancer patients, a case-by-case consideration in selected ambulatory patients receiving chemotherapy is warranted, especially among those with an antecedent history of thromboembolism.

## Conclusions

Patients with advanced NSCLC on platinum-based chemotherapy are predisposed to the development of thromboembolism due to many factors.

Despite the lower incidence of thromboembolism in our study as compared to the quoted retrospective incidences, exclusion of patients with a thrombotic predisposition establishes the incidence of de novo thrombosis in lung cancer patients who are treated with platinum-based chemotherapy, and hence raises a valid question of the need of thromboprophylaxis in a selected group of patients.

## Conflicts of interest

The authors have no conflicts of interest to report.

## Funding statement

This research did not receive any specific grant from funding agencies in the public, commercial and not-for-profit sectors.

## Figures and Tables

**Table 1.** Baseline characteristics of patients.

Baseline Characteristics		Number of patients	Percentages
1.	Age in years		
	Mean	57.5	
	Range	30–79	
2.	Sex		
	Females	27	16.2
	Males	140	83.2
3.	Smoking Habits		
	Smokers(Current /former )	104	62.3
	Never	63	37.7
4.	Histology		
	Adenocarcinoma	61	36.5
	Squamous Cell Carcinoma	106	63.4
5.	Stage		
	2	2	1.2
	3	23	13.8
	4	142	85.0
6.	Baseline Performance Status		
	0	5	3.0
	1	118	70.7
	2	40	24.0
	3	4	2.4

**Table 2.** Thrombosis risk factors.

Thrombosis risk factors		Number of patients	Percentages
1.	Body Mass Index		
	< 18.5 (underweight)	27	16.2
	18.5–24.9 (Normal)	116	69.5
	25-29.9 (Overweight)	22	13.2
	30–34.9 (Class1 obesity )	2	1.2
2.	Diabetes		
	YES	17	10.2
	NO	150	89.8
3.	Hypertension		
	YES	39	23.4
	NO	128	76.6
4.	Hypertriglyceridemia		
	YES	5	3.0
	NO	162	97.0
5.	Use of statins		
	YES	7	4.2
	NO	160	95.8
6.	Baseline Hemoglobin		
	8 ≤ 10 g/dl	16	9.6
	>10 g/dl	151	90.4
7.	Baseline Total leucocyte count (×10^9^/cu mm)		
	≤ 11	103	61.6
	>11	64	38.3
8.	Baseline Platelet Count (×10^9^/cu mm)		
	≤350	110	65.8
	>350	57	34.1
9.	Khorana score		
	Intermediate risk (1–2)	129	77.2
	High risk(>/=3)	38	22.8

**Table 3.** Univariate analysis of risk factors associated with thrombosis.

Variable		No. of patients (%)		*p* value
		NO TE	TE	
1.	Body mass index			
	<18.5 kg/m^2^	25(15.2)	1(0.6)	0.782
	18.5–24.9 kg/m^2^	110(66.7)	5(3.0)	
	25–29.9 kg/m^2^	20(12.1)	2(1.2)	
	>30 kg/m^2^	2(1.2)	0(0)	
2.	Diabetes			
	YES	16 (9.7)	1 (0.6)	0.589
	NO	141(85.5)	7 (4.2)	
3.	Hypertension			
	YES	37(22.4)	2(1.2)	1.0
	NO	120(72.7)	6(3.6)	
4.	Smoking			
	Current/former	101(61.2)	2(1.2)	0.054
	Never	56(33.9)	6(3.6)	
5.	Hypertriglyceridemia			
	YES	5(100)	0(0)	1.0
	NO	152(92.1)	8(4.8)	
6.	Baseline haemoglobin			
	8 ≤ 10g/dl	16(9.7)	0(0)	1.0
	>10 g/dl	141(85.5)	8(4.8)	
7.	Baseline leucocyte count			
	≤ 11000	96(58.2)	6(3.6)	0.712
	>11000	61(37.0)	2(1.2)	
8.	Baseline platelet count			
	≤ 350	104(63.0)	5(3.0)	1.0
	>350	53(32.1)	3(1.8)	
9.	Khorana risk score			
	1–2 (Intermediate risk)	121(73.3)	7(4.2)	0.685
	≥ 3 (High risk)	36(21.8)	1(0.6)	

**Table 4.** Comparison of this study’s results with published data.

	Moore *et al* [11]	Mellema *et al* [13]	This study
Number of patients	204	784	165
Incidence of TEE	11.8%	8%	4.8%
Type of chemotherapy			
Carboplatin combination	None	613	163
Cisplatin combination	204	119	02
Both cisplatin/carboplatin	None	52	None
Timing of thromboembolic events	88% within first 100 days	25% within first 30 days	75% within first 100 days
